# Effects of a Novel, Transdiagnostic, Hybrid Ecological Momentary Intervention for Improving Resilience in Youth (EMIcompass): Protocol for an Exploratory Randomized Controlled Trial

**DOI:** 10.2196/27462

**Published:** 2021-12-03

**Authors:** Anita Schick, Isabell Paetzold, Christian Rauschenberg, Dusan Hirjak, Tobias Banaschewski, Andreas Meyer-Lindenberg, Jan R Boehnke, Benjamin Boecking, Ulrich Reininghaus

**Affiliations:** 1 Department of Public Mental Health Central Institute of Mental Health Medical Faculty Mannheim, Heidelberg University Mannheim Germany; 2 Department of Psychiatry and Psychotherapy Central Institute of Mental Health Medical Faculty Mannheim, Heidelberg University Mannheim Germany; 3 Clinic of Child and Adolescent Psychiatry and Psychotherapy Central Institute of Mental Health Medical Faculty Mannheim, Heidelberg University Mannheim Germany; 4 School of Health Sciences University of Dundee Dundee United Kingdom; 5 Tinnitus Center Charité Universitätsmedizin Berlin Berlin Germany; 6 Centre for Epidemiology and Public Health, Health Service and Population Research Department Institute of Psychiatry, Psychology & Neuroscience King’s College London London United Kingdom; 7 ESRC Centre for Society and Mental Health King’s College London London United Kingdom

**Keywords:** experience sampling methodology (ESM), ecological momentary assessment (EMA), mobile intervention, at-risk individuals, smartphone training, blended care, mental health, stress reactivity, mobile phone

## Abstract

**Background:**

Most mental disorders first emerge in youth and, in their early stages, surface as subthreshold expressions of symptoms comprising a transdiagnostic phenotype of psychosis, mania, depression, and anxiety. Elevated stress reactivity is one of the most widely studied mechanisms underlying psychotic and affective mental health problems. Thus, targeting stress reactivity in youth is a promising indicated and translational preventive strategy for adverse mental health outcomes that could develop later in life and for improving resilience. Compassion-focused interventions offer a wide range of innovative therapeutic techniques that are particularly amenable to being implemented as ecological momentary interventions (EMIs), a specific type of mobile health intervention, to enable youth to access interventions in a given moment and context in daily life. This approach may bridge the current gap in youth mental health care.

**Objective:**

This study aims to investigate the clinical feasibility, candidate underlying mechanisms, and initial signals of the efficacy of a novel, transdiagnostic, hybrid EMI for improving resilience to stress in youth—EMIcompass.

**Methods:**

In an exploratory randomized controlled trial, youth aged between 14 and 25 years with current distress, a broad Clinical High At-Risk Mental State, or the first episode of a severe mental disorder will be randomly allocated to the EMIcompass intervention (ie, EMI plus face-to-face training sessions) in addition to treatment as usual or a control condition of treatment as usual only. Primary (stress reactivity) and secondary candidate mechanisms (resilience, interpersonal sensitivity, threat anticipation, negative affective appraisals, and momentary physiological markers of stress reactivity), as well as primary (psychological distress) and secondary outcomes (primary psychiatric symptoms and general psychopathology), will be assessed at baseline, postintervention, and at the 4-week follow-up.

**Results:**

The first enrollment was in August 2019, and as of May 2021, enrollment and randomization was completed (N=92). We expect data collection to be completed by August 2021.

**Conclusions:**

This study is the first to establish feasibility, evidence on underlying mechanisms, and preliminary signals of the efficacy of a compassion-focused EMI in youth. If successful, a confirmatory randomized controlled trial will be warranted. Overall, our approach has the potential to significantly advance preventive interventions in youth mental health provision.

**Trial Registration:**

German Clinical Trials Register DRKS00017265; https://www.drks.de/drks_web/navigate.do?navigationId=trial.HTML&TRIAL_ID=DRKS00017265

**International Registered Report Identifier (IRRID):**

DERR1-10.2196/27462

## Introduction

### Background

Youth is a critical life period, and most mental disorders have their onset before the age of 25 years [1]. In the early stages of psychopathology, subthreshold expressions of symptoms may occur, reflecting an extended phenotype in the general population that is often transdiagnostic in nature, spanning from subthreshold expressions of anxiety, depression, and mania to psychotic experiences [2-4]. This extended transdiagnostic phenotype may, in turn, be associated with a range of subsequent psychopathological outcomes or exit syndromes later in life [2,3]. On the basis of emerging evidence on the transdiagnostic dimensions of psychopathology [5-10], dimensional classification systems with transdiagnostic high-order spectra that place individuals on a continuum of mental ill-health have recently been proposed, including the Hierarchical Taxonomy of Psychopathology [11-14], and clinical staging models have been proposed considering the overlapping and nonspecific nature of early psychopathology [15-18]. For example, Hartmann et al [16] in their clinical staging model, distinguish three stages of early mental health problems, that is, current psychological distress (stage 1a), a broad Clinical High At-Risk Mental State (CHARMS) with attenuated symptoms of psychosis, mania, or depression (stage 1b), and a first episode of severe mental disorder (stage 2). Moreover, mental disorders in youth aged 10-24 years have been reported to be the leading cause of disease burden in high-income countries [19], underlining the importance of early intervention and prevention. However, access to care remains deficient, with only 1 in 5 youth with mental health problems having access to mental health services [20,21]. Thus, there is a strong need for easily accessible, low-threshold, preventive interventions in the provision of youth mental health services.

Recent rapid advances in digital technologies have led to the development of novel mobile health (mHealth) assessment and intervention techniques, of which ecological momentary assessments (EMAs) [22,23] and ecological momentary interventions (EMIs) [23-28] are, arguably, among the most powerful [26,28]. EMIs such as Acceptance and Commitment Therapy in Daily Life [29,30], recently also referred to as just-in-time adaptive interventions [31], provide a unique opportunity to deliver youth-friendly, adaptive, personalized, real-time transfer of intervention components to individuals’ daily lives. EMIs enable youth to access interventions tailored to what a young person needs in a given moment and context through interactive sampling and administration of specific training components [26,27,32]. To this end, EMIs build on real-time data acquired through EMA, a structured digital diary technique that measures moment-to-moment fluctuations in experience, behavior, and—when coupled with electrocardiography (ECG) and actigraphy sensors—physiological markers in daily life to offer training components that are adapted to the person, moment, and context based on EMA data. Therefore, EMIs are amenable to enhancing access to mental health services for youth depending on their needs and preferences. Indeed, youth—as the generation of digital natives—already make regular use of mHealth apps and are more likely to do so when experiencing psychological distress [33]. However, most mHealth apps that are currently available in major app stores are not evidence-based, often use problematic data sharing and privacy practices, and sometimes contain harmful content [34-36]. As reviewed recently, there is evidence on the effectiveness of mindfulness-based EMI for stress reduction [37] and reduction of psychotic experiences [30,38,39]. Furthermore, there is evidence for higher compliance and greater effectiveness of hybrid interventions that include both digital and face-to-face intervention components with research staff or clinicians [40,41].

Underlying transdiagnostic mechanisms may be important intervention targets in youth to prevent transition to and incidence of severe mental disorder. The most widely studied transdiagnostic mechanisms are (1) elevated stress reactivity (ie, more intense emotional reactions to minor stressors in daily life), (2) heightened interpersonal sensitivity, and (3) enhanced threat anticipation. There is evidence that stress reactivity is elevated in individuals with higher familial or psychometric risk, individuals with an ultrahigh risk state for psychosis, first-episode psychosis, severe and enduring psychosis [42-45], as well as with depressive disorder [46-48], mania, and bipolar disorder [49-51]. Moreover, some evidence suggests that differential reactivity to momentary stressors may reflect a risk and resilience mechanism [44,52-54]. Heightened interpersonal sensitivity is another putative transdiagnostic psychological mechanism that has been characterized by an enduring sense of feeling vulnerable in the presence of others [43,55]. Interpersonal sensitivity has been previously reported as a relevant psychological mechanism in individuals with ultrahigh risk, paranoia, and psychotic disorders [55,56] as well as in individuals with affective disturbances, including depression, anxiety, and bipolar disorder [57-59].

Furthermore, our recent EMA findings extended beyond elevated interpersonal and socioenvironmental sensitivity and, consistent with previous research on psychotic, depressive, and anxiety disorders [60-65], also indicated that enhanced anticipation of threat might be an important mechanism in the development of psychosis [43,44]. These mechanisms have been implicated in a range of adverse mental health outcomes, which we have found to overlap considerably [2,5,6,66] and, as noted above, often manifest at a developmentally early stage in adolescence. Thus, developing EMIs targeting these candidate mechanisms underlying a dimensional transdiagnostic and extended phenotype of psychosis, mania, depression, and anxiety in youth is a promising indicated strategy [67] for preventing transition to, and incidence of, severe mental disorders, which, if effective, will be associated with substantial public health gains.

Compassion-focused interventions are third-wave cognitive behavioral therapy (CBT) approaches that use a wide range of innovative therapeutic techniques for enhancing emotional resilience by activating emotion regulation systems related to self-compassion, self-acceptance, and positive affect rather than those related to stress, threat, anxiety, and depression [68-70]. Indeed, there is meta-analytic evidence on compassion-focused interventions treating various conditions [71-73], such as depression and anxiety [74], psychosis [75], and general distress [76]. Compassion-focused interventions involve the use of techniques that seek to access emotion regulation processes through imagery rather than rational understanding [68,69]. In doing so, compassion-focused interventions aim to enhance emotional resilience by developing specific affective regulation patterns and, thereby, reduce reactivity to stress, hypervigilance for threat, interpersonal sensitivity, and negative affective appraisals. Experimental evidence indicates that compassion-focused intervention techniques can reduce negative affect and paranoia in moments of high stress [77,78]. Therefore, compassion-focused interventions are particularly promising for targeting these putative transdiagnostic mechanisms. Building on these pieces of evidence, we have recently developed a novel, accessible, transdiagnostic, compassion-focused, hybrid intervention to enhance resilience in youth with early mental health problems—the EMIcompass intervention [53], which consists of an EMI plus face-to-face training sessions. Although there is preliminary evidence on the feasibility and initial therapeutic effects of the EMIcompass intervention from an uncontrolled pilot study [53], robust evidence on the underlying mechanisms, feasibility, and initial signals of efficacy of EMIcompass from an exploratory trial is pending.

### Objectives

Against this background, this study will aim to examine the clinical feasibility, underlying mechanisms, and initial signals of efficacy of EMIcompass for improving resilience in an exploratory, randomized controlled trial (RCT) of youth with current psychological distress, a broad CHARMS, or a first episode of severe mental disorder. The EMIcompass intervention will be administered in addition to treatment as usual (TAU) in the experimental condition compared with a control condition of TAU only. Specifically, this study’s aims are as follow:

1. to establish the clinical feasibility of the trial methodology and deliver the EMIcompass intervention to youth with early mental health problems (based on successful recruitment, assessment of inclusion criteria, randomization, retention in the assessment of outcomes, fidelity of delivering the intervention, compliance with the intervention protocol, satisfaction, and acceptability);
2. to detect initial signals of the efficacy of the EMIcompass intervention in reducing psychological distress (candidate primary outcome), primary (ie, psychotic, manic, anxiety, or depressive) symptoms, and general psychopathology (candidate secondary outcomes) at postintervention and 4-week follow-up;
3. to test the effects of the EMIcompass intervention on reducing stress reactivity (primary candidate mechanism), threat anticipation, interpersonal sensitivity, negative affective appraisals, resilience, self-compassion, emotion regulation, and physiological markers of stress reactivity (secondary candidate mechanisms) at postintervention and 4-week follow-up; and
4. to explore whether the effects of the EMIcompass intervention on psychological distress, primary (ie, psychotic, manic, anxiety, or depressive) symptoms, and general psychopathology are mediated via pathways through stress reactivity, threat anticipation, interpersonal sensitivity, negative affective appraisals, resilience, self-compassion, and emotion regulation.

## Methods

### Study Design

**Figure 1:**
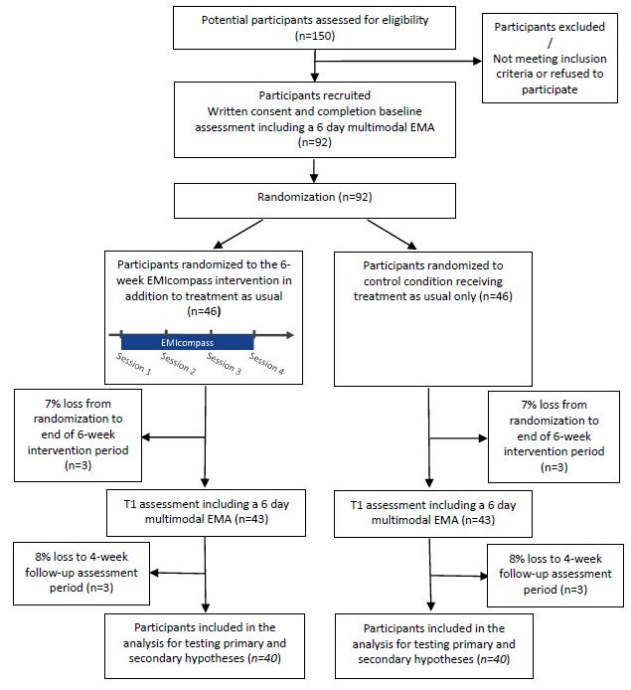
Study flowchart. EMA: ecological momentary assessment, collected eight times per day on 6 consecutive days (including self-reported and activity or electrocardiography sensor); n denotes the total number of participants.

In an exploratory RCT, youth aged 14-25 years will be randomly assigned to the EMIcompass intervention in addition to TAU (experimental condition) or a control condition of TAU only, which will include routine mental health care. Participants will be recruited from mental health services in Mannheim, Germany, and via advertisements on the institute’s webpage, Facebook, and Instagram and via local registries. Candidate mechanisms and outcomes will be assessed before randomization (at *baseline*), at the end of the 6-week intervention period (*postintervention*), and at the 4-week follow-up (ie, 4 weeks after completing the intervention period) by blinded assessors (Figure 1). Randomization will be conducted by an independent researcher using a computer-generated sequence. The assessment of outcomes and statistical analyses will be blinded to the treatment allocation.

### Participants

We will recruit and randomize 92 individuals with current psychological distress, CHARMS or a first episode of severe mental disorder based on a modified version of the clinical staging model by Hartmann et al [16]. Individuals presenting to mental health services at the Central Institute of Mental Health (CIMH), Mannheim, will be approached by a clinician who will provide initial information about the study. If the individual agrees, their treating clinician will pass on their contact details to the research team. In addition, individuals from the general population, who do not seek help from mental health services at CIMH, will be recruited for example, via social media or local registries. All participants will then be contacted by the research team, and initial information about the study will be provided. Following this, individuals will be fully informed about the study, and written informed consent will be obtained by researchers with a master’s degree in psychology (including parents or legal guardians for minors), which can be withdrawn at any time without any negative consequences. Eligibility will then be assessed in an interview using observer-rated measures (Structured Clinical Interview for Diagnostic and Statistical Manual of Mental Disorders, fifth edition (DSM-5) [79]; Comprehensive Assessment of At-Risk Mental State [80] completed by the researcher; and self-reported measures using the Kessler Psychological Distress Scale [1,81]). All participants will be reimbursed for their time and travel expenses at the end of the study.

### Ethics Approval and Consent to Participate

The study, titled *Efficacy of a novel, accessible, transdiagnostic, compassion-focused ecological momentary intervention for help-seeking youth (EMIcompass)*, has received ethical approval from the local ethics committee of the Medical Faculty Mannheim Heidelberg University (2017-602N-MA, date: September 7, 2017). Ethical approval was granted before funding was obtained, given that this is a requirement for project grants by the German Research Foundation. All participants and, in the case of individuals aged <18 years, parents or legal guardians will provide written informed consent before inclusion in the study. The sponsor has an insurance, which covers accidents on the journeys to the study appointments. However, no insurance covers harm from study participation, as this is expected to be of low risk.

### Inclusion and Exclusion Criteria

Textbox 1 provides an overview on the inclusion and exclusion criteria, and Table 1 defines the inclusion criteria in more detail.

**Inclusion and exclusion criteria.**

**Inclusion criteria**

- Age between 14 and 25 years
- Meeting criteria for one of the following stages (based on a modified version of the clinical staging model by Hartmann and colleagues [16]): individuals with current psychological distress (stage 1a), that is, a score of 20 or above on the Kessler Psychological Distress Scale [1,81], but no Clinical High At-Risk Mental State (stage 1b) or first episode of severe mental disorder (stage 2); individuals who meet criteria for a Clinical High At-Risk Mental State (stage 1b); individuals, who meet criteria for a first episode of psychotic disorder, bipolar disorder, severe depressive disorder or severe anxiety disorder according to according to the Diagnostic and Statistical Manual of Mental Disorders, fifth edition (DSM-5) (stage 2)
- High emotional reactivity assessed with a two-item self-report measure (instruction: “Please think of the most unpleasant event in the last week: (1) How sad, disappointed or angry have you been? (2) Have you been sad, disappointed or angry because of your feelings?”) rated on a 7-point Likert scale (ie, a score of ≥3 indicating high reactivity) or by the interviewer-rated Comprehensive Assessment of At-Risk Mental State subscale [80] rated on a 7-point Likert scale (ie, with a rating of ≥3 indicating high reactivity)
- Reduced positive affect (ie, a mean positive affect score below 3.19 for men and 3.05 for women based on normative scores from a representative sample of the German population [82]) or increased negative affect (ie, a mean negative affect score above 1.81 for men and 1.75 for women [82]) assessed using the Positive Affect and Negative Affect Scale [83]
- Willingness to participate in the EMIcompass intervention
- Ability to provide written informed consent (or consent by parents in the case of minors)

**Exclusion criteria**

- A primary diagnosis of alcohol or substance abuse or dependence, assessed using the Structured Clinical Interview for DSM-5 [79]
- Evidence that symptoms are precipitated by an organic disease
- Insufficient command of German so that the intervention cannot be followed, and outcomes cannot be reasonably assessed in German
- Diagnosis of a learning disability according to case records
- Current suicidal ideation (indicated by a score>4 in the Comprehensive Assessment of At-Risk Mental State [80])

**Table 1:** Inclusion criteria and transdiagnostic sample characteristics based on a modified version of the clinical staging model by Hartmann et al [16].

Stage and criteria					Measure
**1a (distressed individuals)**					
			Psychological distress (K10^a^ score ≥20) but not fulfilling criteria of stage 1b or 2		K10
**1b (CHARMS^b^)**					
	**Psychosis trait vulnerability**				
		First degree relative with psychosis and SOFAS^c^ < 50 in the last 12 months or Or SOFAS 30% below the past level		Family risk SOFAS	
	**Psychosis trait vulnerability**				
		Schizotypal personality and SOFAS <50 in the last 12 months or Or SOFAS 30% below the past level		SCID II^d^ SOFAS	
	**Bipolar trait vulnerability**				
		Depressed mood or diminished interest or pleasure for at least 1 week as well as two additional criteria of depression: weight loss, sleep disorder, psychomotor disturbances, loss of energy, feelings of worthlessness or guilt, diminished ability to think or concentrate or indecisiveness, suicidality And mood swings for at least 6 months in the lifetime (not symptom-free for a longer period than 2 months consecutively) and at least three symptoms: decreased need for sleep, increased energy, inflated self-esteem or grandiosity, increase in goal-directed activity, restlessness, increased talkativeness, unusual ideas, risky behavior, inappropriate humor (does not have to equal loss of function!) Or first degree relative with bipolar disorder		SCID-5^e^ Family risk	
	**Attenuated psychotic symptoms**				
		CAARMS^f^ global rating score of 3-6 and frequency of 3-6 on the subscales: unusual thought content, nonbizarre ideas, perceptual abnormalities, disorganized speech Or global rating score of 6 and frequency of 3 on the subscales: unusual thought content, nonbizarre ideas, perceptual abnormalities, disorganized speech		CAARMS	
	**Attenuated hypomanic symptoms**				
		Elevated, expansive or unusually irritable mood on at least 2 consecutive days And 2 (or in case of only irritable mood 3) additional criteria: inflated self-esteem or grandiosity, decreased need for sleep, increased talkativeness, flight of ideas or subjective experience that thoughts are racing, distractibility, increase in goal-directed activity or psychomotor agitation, unusual ideas, increased involvement in activities that are pleasurable in short time but have a high potential for long-term damage For a duration of 3 days maximum if 3 or more (or in case of only irritable mood 4 or more) additional criteria are met and there are functional disturbances or others notice the mood or functional disturbances For a duration of 6 days maximum if 3 or more (or in case of only irritable mood 4 or more) additional criteria are met or there are functional disturbances or others notice the mood or functional disturbances Exclusion: hospitalization, severe impairment in social or professional functioning, no psychotic elements		CAARMS	
	**Moderate (attenuated) depression**				
		Mild or moderate depression (current or lifetime), that is, at least 1 cardinal symptom, 5 additional symptoms And HAM-D^g^>17 (cutoff)		SCID-5 HAM-D	
	**BLIPS^h^**				
		Global rating of 6 on the subscales: unusual thought content or nonbizarre ideas Or global rating of 5 or 6 on the subscale perceptual abnormalities And/or global rating of 6 on the subscale disorganized speech present for less than a week And frequency of 4-6 on all above mentioned scales		CAARMS	
	**Anxiety**				
		Mild or moderate panic disorder /agoraphobia (current or lifetime) Or not fully meeting criteria for GAD^i^, that is, symptoms for less than 6 months or less than four symptoms met Or mild or moderate social phobia (current or lifetime) And HAM-A^j^>9 (cutoff)		SCID-5 HAM-A	
**2 (first treated episode)**					
	Psychosis				CAARMS
	Severe major depression (current or lifetime)				SCID-5
	Mania or hypomania				SCID-5
	Severe anxiety disorder (current or lifetime); eg, agoraphobia, GAD				SCID-5

^a^K10: Kessler Distress Scale [81].

^b^CHARMS: Clinical High At-Risk Mental State.

^c^SOFAS: Social and Occupational Functioning Assessment Scale [84].

^d^SCID II: Structured Clinical Interview for DSM-IV Axis II Personality Disorders,

^e^SCID-5: Structured Clinical Interview for Diagnostic and Statistical Manual of Mental Disorders, fifth edition (DSM-5) [79].

^f^CAARMS: Comprehensive Assessment of At-Risk Mental State [80].

^g^HAM-D: Hamilton Depression Rating Scale [85].

^h^BLIPS: brief limited intermittent psychotic symptoms.

^i^GAD: Generalized Anxiety Disorder.

^j^HAM-A: Hamilton Anxiety Rating Scale [86].

### Interventions

#### Control Condition: TAU

Participants allocated to the TAU control condition will continue to receive all the treatment they received before the start of the study. This will include good standard care delivered according to local and national service guidelines and protocols by their general practitioner, psychiatrist, and other providers of (mental) health care. Service contacts will be assessed for the duration of the trial using the Client Service Receipt Inventory [87] to monitor variation in the delivery of, and engagement with, mental health services.

#### Experimental Condition: EMIcompass Intervention Plus TAU

The EMIcompass intervention will be delivered by trained psychologists within a 6-week period in addition to TAU to individuals allocated to the experimental condition. TAU consists of all the treatment individuals received before inclusion in the study, including their general practitioner, psychiatrist, and clinical psychologist, except for treatment using elements of third-wave CBT. The manualized EMIcompass intervention consists of four biweekly sessions (three training sessions and one review session) with a duration of 45-60 minutes administered face-to-face or using a certified and encrypted video conferencing system and a 6-week compassion-focused EMI. An optional on-demand session will be offered if participants are unable to complete tasks between sessions or report acute psychological distress so that a scheduled session cannot be followed as per the manual. The EMI, which translates the training from the intervention sessions into individuals’ daily lives, will be administered through a smartphone-based app (movisensXS, movisens GmbH) running on dedicated study smartphones. The first three sessions are based on elements of compassion-focused therapy [68]. In line with our pilot study [53], compassion-focused therapy principles and techniques are introduced in these guided sessions. The first session aims to familiarize the participants with the app offering EMI. Further, practical tasks to activate the soothing system, as a key emotion regulation system in compassion-focused therapy, are presented and trained together with the psychologist, as described elsewhere [88]. Face-to-face sessions also offer the opportunity to reflect on the progress and problems participants face with EMI. In the last session, progress with all tasks will be reviewed and subjective improvement in, compliance and satisfaction, and acceptance of the EMIcompass intervention will be assessed.

The app will offer EMI tasks according to three types of delivery schemes: (1) enhancing, (2) consolidating, and (3) interactive EMI tasks that aim for ecological translation of therapeutic principles and techniques to daily life. Participants will be asked to complete one *enhancing task* per week, practicing new compassion-focused tasks such as self-compassionate writing, experiencing emotions as a wave, and discovering their own compassionate self. Furthermore, participants will be offered *consolidating tasks* covering components of *enhancing tasks* from previous days. The components of *consolidating tasks* will be extended each time an *enhancing task* has been presented until all components are covered. In addition, participants will be offered to complete a brief EMA of momentary stress, affective disturbance, and threat anticipation six times per day, 3 days per week. On the basis of these EMAs, *interactive* tasks will be offered if participants score high on momentary stress or negative affect. The threshold for triggering interactive tasks will be either high momentary negative affect operationalized as a score of 4 or higher (on a 7-point Likert scale ranging from 1-7) on items of established and validated EMA measures of negative affect (Textbox 2) or high momentary stress based on items of established and validated measures of EMA of event-related, activity-related, or social stress (ie, a score <0 on a bipolar scale ranging from −3 to 3).

Participants will be instructed in detail in the semantic meaning of the 7-point Likert and bipolar scales and encouraged to carefully observe moment-to-moment variation in, and make use of the full range when rating, these scales on EMA items that have been previously used and validated to measure moment-to-moment variation in stress or negative affect. Given that a crucial element of compassion-focused therapy is for individuals to use compassionate imagery in moments of high stress or negative affect, these interactive tasks reflect an important component of the EMIcompass intervention. Participants can decline the EMI tasks in each delivery scheme. After completing the intervention period, participants will return the study devices and will no longer have access to the app.

**Ecological momentary assessment domains and measures.**

**Momentary stress**

Momentary stress was operationalized as unpleasant events, activities, and social situations in daily life. In line with previous research, we distinguished three different types of stress, that is, event-related stress, activity-related stress, and social stress [43,89]. Participants will be asked to report the most important event that happened in the time since the last assessment. This event will be subsequently rated on a 7-point Likert scale (−3=very unpleasant, 0=neutral, 3=very pleasant) comprising the event-related stress measure. Activity-related stress will be assessed by asking participants to report their current activity and then judge the valence of the activity (“This is...”) using a 7-point Likert scale (1=very unpleasant, 7=very pleasant). Further, social stress will be measured by asking participants to evaluate the social context when other people were present as well as when they were alone by answering the items “I am taking care of myself”/“I am taking care of somebody” and “I would rather be alone”/“I would prefer to have company” using two 7-point Likert scales and the item on social valence (“This is...” rated on the 7-point Likert scale from 1=very unpleasant to 7=very pleasant)

**Negative affect**

Six items will be used to assess negative affect (anxiousness, loneliness, insecurity, anger, annoyance, and feeling down), using 7-point Likert scales ranging from “not at all” (rating of 1) to “very much” (rating of 7)

**Negative affective appraisals**

In case participants report negative affect (ratings >3), two items on how they cope with their negative affect will be displayed: “I want to change my negative feelings” and “I would like to get rid of my negative feelings” using two 7-point Likert scales

**Positive affect**

Positive affect will be assessed by four items (cheerfulness, satisfaction, enthusiasm, and feeling relaxed) using 7-point Likert scales ranging from “not at all” (rating of 1) to “very much” (rating of 7)

**Aberrant salience**

The ecological momentary assessment measure comprises three items in line with [43]. The items will be rated on a 7-point Likert scale (ranging from 1 [“not at all”] to 7 [“very much”]): “Everything grabs my attention right now,” “Everything seems to have meaning right now,” and “I notice things that I haven’t noticed before”

**Self-esteem**

Three items will be rated on a 7-point Likert scale ranging from “not at all” (rating of 1) to “very much” (rating of 7): “I feel guilty,” “I doubt myself,” “I feel disappointed about myself”

**Self-compassion**

Three items will be rated on a 7-point Likert scale ranging from “not at all” (rating of 1) to “very much” (rating of 7): “I like myself,” “I feel safe,” “I feel benevolent”

**Psychotic experiences**

Psychotic experiences will be assessed using eight items on thought problems and hallucinations (“I see things that aren’t really there,” “I hear things that aren’t really there,” “I feel suspicious,” “It's hard to express my thoughts in words,” “I feel unreal,” “My thoughts are influenced by others,” “I can’t get these thoughts out of my head,” “I feel like I am losing control”) that will be rated on 7-point Likert scales ranging from “not at all” (rating of 1) to “very much” (rating of 7)

**Resilience**

If participants indicate that there was a negative event (valence −3 or −2), then the item “I had difficulties to recover” will be rated on a 7-point Likert scale

**Threat anticipation**

In line with previous research, we will ask participants to rate the likelihood of negative events happening to them in the future [43]. They will be asked to think of what might happen in the next few hours and to rate the item “I think that something unpleasant will happen” on a 7-point Likert scale from “not at all” (rating of 1) to “very much” (rating of 7)

**Disturbance**

“This prompt disturbed me” will be rated at the end of each assessment on a 7-point Likert scale from “not at all” (rating of 1) to “very much” (rating of 7)

### Clinical Feasibility, Acceptability, Treatment Adherence, and Intervention Fidelity

Clinical feasibility will be assessed in relation to the trial methodology and the delivery of the EMIcompass intervention to youth with early mental health problems. The feasibility of the trial methodology will be assessed based on the following criteria: (1) successful recruitment of at least 96 participants during the study period; (2) assessment of inclusion criteria in 95% of potential participants after obtaining written consent; (3) successful randomization of at least 92 participants after completion of eligibility and baseline assessment; and (4) a retention rate of at least 85% for assessment of outcomes at least at one of the two time points at postintervention and 4-week follow-up. In addition, the following criteria will be used for establishing the feasibility of delivering the EMIcompass intervention, including its acceptability, intervention adherence, and intervention fidelity: (1) satisfaction with the EMIcompass intervention in general, ease of use, accessibility and comprehensiveness of various components of the intervention in a debriefing questionnaire [30,90], and the subjective quality of EMIcompass using the mobile application rating scale [91]; (2) compliance with, and adherence to, the EMIcompass intervention protocol based on a satisfactory level of session attendance, an adherence checklist covering all core components [29,30,92], and adherence to EMI tasks (ie, mean number of consolidating/interactive EMI tasks completed per week); and (3) fidelity to the EMIcompass intervention protocol based on independent ratings of a random selection of audio recordings of three face-to-face sessions, including fidelity to session protocol (ie, independent rating of core components delivered by trained psychologist), ability to model and embody the spirit of compassion, and the use of microskills in compassion-focused therapy assessed by the Compassion Focused Therapy-Therapist Competence Rating Scale [93].

### Candidate Mechanisms and Outcomes

#### Overview

After obtaining written informed consent, all eligible participants will be assessed on candidate mechanisms and outcomes before randomization (*baseline*, t_0_), after the 6-week intervention period (*postintervention*, t_1_) and after a 4-week follow-up period (*follow-up*, t_2_) by blinded assessors (Figure 1 and Multimedia Appendix 1). Research Electronic Data Capture (REDCap) [94], a secure, web-based software platform hosted at the CIMH servers, will be used for data collection.

#### Primary Candidate Mechanism

The primary candidate mechanism is a reduction in stress reactivity acquired by EMA from baseline to postintervention for the experimental condition compared with the control condition. EMA will include eight assessments per day, scheduled at random within set blocks of time, for 6 consecutive days at baseline, postintervention, and follow-up [43,95]. Momentary stress will be assessed using established and validated EMA measures of event-related stress, activity-related stress, and social stress (Textbox 2) [43,89]. We will compute a composite momentary stress measure (ie, the mean score of event-related, activity-related, and social stress) in line with the literature [52,96,97] and the EMIcompass pilot study [53]. Negative affect will be assessed using an established and validated EMA measure of negative affect [43]. Stress reactivity as the primary candidate mechanism will be computed in linear mixed models with composite momentary stress as the independent variable and negative affect as the outcome variable [43,89,95].

#### Secondary Candidate Mechanisms

Secondary candidate mechanisms (Multimedia Appendix 1 and Textbox 2) measured using EMA include threat anticipation, negative affective appraisals, emotional resilience to stress (operationalized as attenuated recovery in positive affect in response to minor stressors), and elevated stress reactivity (ie, increased negative affect) in response to event-related, activity-related, and social stress using subscale scores of the EMA stress measure. Threat anticipation will be additionally assessed using the Threat Anticipation Measure [62], asking participants to estimate the future likelihood of a list of negative, neutral, and positive events happening to themselves and other people [61-63]. Interpersonal sensitivity will be assessed using the Interpersonal Sensitivity Measure [98] in addition to EMA. Resilience will be measured using the Connor-Davidson Resilience Scale [99] and the Resilience Scale [100]. Furthermore, the Self-Compassion Scale [101], the Fife Facet Mindfulness Questionnaire [102], and the Cognitive Emotion Regulation Questionnaire [103] will be used to assess self-compassion and emotion regulation. In addition, we will assess physiological markers of stress reactivity using a sensor for ambulatory ECG and actigraphy (movisens ECGmove4) during the 6-day EMA at baseline, postintervention, and at follow-up.

#### Candidate Primary Outcome

The candidate primary outcome of this exploratory RCT is psychological distress measured using the well-validated Kessler Psychological Distress Scale [81]. The 10 items are rated on a 1 (never) to 5 (always) Likert scale focusing on psychological distress in the last month. Strong psychometric properties have been reported with a reliability of Cronbach α>.90 [81].

#### Candidate Secondary Outcomes

Secondary outcomes include primary (ie, psychotic, manic, anxiety, or depressive) symptoms and general psychopathology. These will be assessed using the following observer-rated measures: the Brief Psychiatric Rating Scale [104], including the Comprehensive Assessment of At-Risk Mental State [80], the Young Mania Rating Scale [105], the Hamilton Depression Rating Scale [85] and the Hamilton Anxiety Rating Scale [86]. On the basis of these measures, we will assess the transition to another clinical stage (according to a modified version of the clinical staging model by Hartmann et al [16]; see above). In addition, the following self-report measures will be used: the Brief Symptom Inventory [106], the Beck Depression Inventory [107], and the Prodromal Questionnaire [108]. Secondary outcomes further include quality of life measured using the WHO-Quality of Life assessment [109].

### Other Measures

Other study parameters will include basic sociodemographic characteristics, familial risk factors for psychopathology, and other parameters (including age, sex, alcohol or substance use, and childhood trauma [110]). The Client Service Receipt Inventory [87] will be used to record patients’ contacts with mental health services, monitor variation in the delivery of TAU, and model economic outcomes for a definitive trial. The Working Alliance Inventory [111,112] will be used to assess the relationship between practitioners and patients.

### Sample Size

A formal sample size calculation is not essential for this exploratory trial, which primarily seeks to establish feasibility, effects on candidate mechanisms, and initial signals of efficacy. In planning, we aimed to determine the sample size in such a way as to establish the feasibility of the methodology for conducting an RCT and delivering the EMIcompass intervention to youth with early mental health problems and initial signals of the efficacy of EMIcompass in reducing psychological distress as a candidate primary outcome (see Statistical Analysis Plan [113] for further detail) as a basis for a future definitive trial. For the latter, previous studies of third-wave CBT [72,114], including compassion-focused interventions [68,77], suggest that these types of interventions may yield clinically meaningful reductions in psychological distress of moderate to large effect size. This is consistent with the initial findings from an uncontrolled phase I pilot study [53]. However, even if the effect size for the main effect of condition on psychological distress (primary outcome) in this exploratory RCT is small, a power simulation in the R environment indicated that a sample size of N=80 participants (40/40, 50% experimental, 40/40, 50% control condition) would be sufficient to detect a small effect size of 0.3 across the postintervention 4-week follow-up with a power of 81% when testing at α=.05 for the effect of condition (experimental vs control condition) on psychological distress using linear mixed modeling, which will be tested using a Wald-type test of no difference between the two conditions across both time points against the two-sided alternative hypothesis that the conditions are, on average, different across the two follow-up time points (given the exploratory nature of this trial), while controlling for baseline psychological distress and group status. At the 4-week follow-up, we expect an attrition rate of 15%, resulting in a loss to follow-up of approximately 6 individuals per condition on average (Figure 1). Therefore, we will randomize a total of 92 participants at baseline, leaving 80 participants at follow-up to detect a small effect size of 0.3 at this time point. This sample size is also sufficient to test the criteria for establishing feasibility. Simulation studies on power and accuracy for multilevel mediation models with continuous variables [115] and our recently completed multilevel moderated mediation analysis of EMA data [42] suggest very little bias in parameter estimates with samples of this size (and 40 repeated measures, on average, per participant).

### Randomization and Blinding

Participants will be randomized at a 50:50 ratio to the experimental or control condition at the level of the individual participant after completion of the baseline assessment. Block randomization in blocks of four will be performed by an independent research assistant through a computer-generated sequence, with stratification for the three stages (ie, stages 1a, 1b, and 2). The assessors will be blind to the allocation of participants when assessing outcomes at postintervention and follow-up. Any data specific to the intervention group (eg, clinical feasibility) will be stored in a separate database. Breaks in masking will be documented, and another (blinded) researcher will repeat the assessment to maintain masking.

### Assessment of Safety

Serious adverse events (SAEs) will be monitored throughout the entire study period and reported to the accredited Medical Ethics Review Committee, the Data Monitoring and Ethics Committee (DMEC), and, where required, the Trial Steering Committee (TSC). SAEs are any serious incidents that result in death, persistent or significant disability or incapacity that require hospitalization, or life-threatening situations. SAEs are not expected to occur as a result of the intervention. If there are doubts about safety or ethical concerns, the TSC will terminate the trial. The DMEC will advise on safety and ethical concerns, monitor evidence for harm by the intervention (eg, SAEs) in the experimental condition, and review whether these events are in line with expectations. If deemed necessary, the DMEC can recommend to the principal investigator (PI) and TSC for interim analyses to be conducted and the trial to be terminated prematurely.

### Statistical Analysis

The primary objective of this exploratory RCT is to establish the feasibility of the trial methodology and intervention delivery and initial signals of efficacy on the candidate primary outcome (ie, psychological distress) as a basis for a future definitive trial. In addition, this trial seeks to obtain parameter estimates (95% CI) for the effects on primary and secondary candidate mechanisms and candidate secondary outcomes. A detailed Statistical Analysis Plan [113] has been agreed with the DMEC and the TSC and has been preregistered and published on the Open Science Framework. It was registered while collecting the data before study completion and accessing the locked database. Descriptive statistics will be used, and CIs will be constructed as appropriate to compute basic sample characteristics and address the primary aim of establishing the feasibility of the trial methodology and intervention delivery based on the criteria described above and in further detail in the Statistical Analysis Plan using three categories (in line with a traffic light system): (1) feasibility fully established (green), (2) feasibility established, but study procedures need to be modified (yellow), and (3) feasibility not established (red) [113]. The analysis of candidate mechanisms and initial signals of efficacy has been described in detail in the Statistical Analysis Plan [113] and will be an intention-to-treat analysis or an available case analysis following intention-to-treat principles, with data from all participants entered into the analysis, including those who have low adherence to or who will drop out from the intervention. We will make every effort to assess all participants at postintervention and 4-week follow-up. Linear mixed modeling in Stata 16 will be used to compare candidate mechanisms and outcomes between experimental and control conditions at postintervention and 4-week follow-up. The primary candidate outcome of psychological distress measured at postintervention and 4-week follow-up will be entered as the dependent variable and psychological distress measured at baseline, group status (3-level factor), time (2-level factor), and condition (2-level factor) as independent variables. The main effect of condition on psychological distress will be parameterized so that it reflects the difference between the two conditions at the two follow-up time points (ie, postintervention and 4-week follow-up), which will be tested (at α=.05) by a Wald-type test with df=1, which tests the joint null hypothesis of no difference at both follow-up time points against the alternative hypothesis that there is, on average, a difference across the two follow-up time points. In addition, given the exploratory nature of this trial, with the main goal of establishing feasibility and obtaining parameter estimates for a future definitive RCT, 95% CI for the two time-specific contrasts of a time×condition interaction term will be inspected to obtain estimates for the differences across conditions at each of the two time points, with a time × condition interaction and a baseline psychological distress×time interaction added as independent variables to the previous model. Within-subject clustering of repeated measures (postintervention and 4-week follow-up) will be taken into account by including a level-2 random intercept and allowing the models’ level-1 residuals to be correlated with a completely unstructured error variance-covariance matrix. The model will be fitted using restricted maximum likelihood estimation. The analysis of secondary candidate outcomes and primary and secondary candidate mechanisms will, in principle, follow the same steps, focusing on 95% CIs (rather than P values at α<.05). Multilevel moderated mediation analysis of EMA data will be used to explore whether the effects of condition on primary (ie, psychotic, manic, anxiety, or depressive) symptoms are mediated via stress reactivity, threat anticipation, negative affective appraisals, and interpersonal sensitivity [42]. As participants will be randomly assigned to experimental and control conditions, no differences across conditions are expected in other study parameters (sociodemographic characteristics, alcohol or substance use, and childhood trauma). No statistical tests will be performed on these study parameters at baseline.

## Results

### Overview

The trial is ongoing. It started recruitment on July 15, 2019, and the first enrollment was conducted in August 2019. We are currently working with trial protocol version 5 (June 24, 2020). The last changes to the protocol were related to adaptations because of the COVID-19 pandemic, such as introducing the option of using video conferencing systems. As of May 2021, enrollment and randomization were completed (n=92 participants). Assessment of outcomes at postintervention and follow-up is still ongoing, with the last assessment for the last participant being scheduled for August 2021. Data will then be entered, checked, and the database locked (by September 2021). We expect results to be published in 2022.

### Research Governance

The CIMH is the trial sponsor. The study has received ethical approval by the local ethics committee (EC) of the Medical Faculty Mannheim, Heidelberg University (2017-602N-MA). Amendments to the study protocol will be submitted to the EC and sent to the DMEC, TSC, and study sponsor. The trial is registered at the clinical trial register, and changes to the protocol will be updated. Deviations from the protocol will be documented in the study folder using a breach report form and will be reported to the TSC. The trial does not involve the collection or storage of biological samples. All data will be handled confidentially and will be coded using a number according to the order of study entry. Data will be securely stored in line with the European General Data Protection Regulation. Personal data will be kept separately from pseudonymized data. The PI has overall responsibility for the trial. The trial research team will meet regularly and will be chaired by the PI. It will manage the day-to-day running of the study, monitor the progress of the trial (ie, recruitment and assessment), and oversee the preparation of presentations and reports to EC, TSC, and DMEC. The TSC will meet biannually and provide independent overall supervision, monitor the progress of the trial (eg, recruitment, data completion rates, and adherence to the protocol), and approve the protocol and any amendments. The DMEC will meet at least once per year, advise on ethical or safety concerns, monitor SAEs and other evidence of intervention harm and whether this is in line with expectations. If deemed necessary, the DMEC can recommend that the PI and TSC are granted access to all trial data, to perform interim analyses and to terminate the trial prematurely.

## Discussion

Transdiagnostic mechanisms implicated in the development of severe mental disorders are important targets for prevention and early intervention. Ecological translation of compassion-focused intervention components to individuals’ daily lives through an EMI offers new avenues for tangible prevention strategies delivering real-world and real-time interventions that are easily accessible by youth [36]. Findings from a recent, nationally representative survey suggest that psychological distress, social isolation, lack of company, and worrying during the COVID-19 pandemic were highly prevalent in youth and, interestingly, associated with the current use of and a positive attitude toward digital interventions [33]. EMIs are also amenable to enhancing access to mental health services for youth depending on their needs and preferences, for instance, by delivering low-threshold interventions by frontline mental health staff [116-118] as a component that can be rolled out across adolescent and adult mental health services and link in with what is urgently needed, that is, a wider youth mental health reform that aims to provide seamless coverage of mental health care with smooth transitions from adolescence to mature adulthood at an age of approximately 25 years [20]. Furthermore, EMIs allow for investigating the strength of the evidence in support of several causal criteria (ie, association, temporality, sole plausibility, and ecological validity) as part of the ecological interventionist causal model approach that targets candidate underlying psychological mechanisms in daily life to achieve sustainable change under real-world conditions [27]. However, robust, trial-based evidence on EMIs and other mHealth interventions remain very limited [26,27,29,36,116,119]. A key next step is to examine the efficacy of youth-friendly, accessible, interactive, real-time interventions targeting candidate mechanisms underlying the transdiagnostic phenotype of psychosis, mania, depression and anxiety and thereby, help preventing adverse outcomes later in life. While, in the current study, we use EMA items that have been previously used and validated to measure moment-to-moment variation in stress/negative affect, with considerable within-person variability having been observed for these items in several EMA studies [43,89,120], inter-individual differences in within-person variability as well as general response tendencies may influence the number of triggered EMI tasks and hence, further research is needed to optimize and personalize the assignment of EMI components, eg, by using methods of artificial intelligence, recurrent neural networks (RNNs) in particular [121]. For example, we currently aim to apply recurrent neural networks in an ongoing study of personalized digital mental health promotion and prevention in youth [33]. In addition, clinical staging models of severe mental disorders require further scrutiny, including heterogeneity in phenomenology, course, and outcome within individual stages [5,122,123].

The present exploratory RCT is the first to establish feasibility, evidence on underlying mechanisms, and preliminary signals of efficacy of a compassion-focused, hybrid EMI for reducing stress reactivity (EMIcompass) in youth at different clinical stages. Preliminary evidence from a pilot study of the EMIcompass intervention in help-seeking youth showed reductions in clinical symptoms and stress reactivity [53]. If this exploratory trial is successful, a confirmatory RCT will be warranted. Overall, our approach has a scalable potential to prevent the transition of early mental health problems to severe and enduring mental disorders not only in individuals at risk of developing psychosis but transdiagnostically and across clinical stages and, thereby, significantly advance preventive interventions in youth mental health provision.

## Appendix

Multimedia Appendix 1 Standard Protocol Items: Recommendations for International Trials (SPIRIT) figure.

## Abbreviations

CBT: cognitive behavioral therapy
CHARMS: Clinical High At-Risk Mental State
CIMH: Central Institute of Mental Health
DSM-5: Diagnostic and Statistical Manual of Mental Disorders
DMEC: Data Monitoring and Ethics Committee
EC: ethics committee
ECG: electrocardiography
EMA: ecological momentary assessment
EMI: ecological momentary intervention
mHealth: mobile health
PI: principal investigator
RCT: randomized controlled trial
REDCap: Research Electronic Data Capture
SAE: serious adverse event
TAU: treatment as usual
TSC: Trial Steering Committee
