# Supplementary material for: Effects of a Novel, Transdiagnostic, Hybrid Ecological Momentary Intervention for Improving Resilience in Youth (EMIcompass): Protocol for an Exploratory Randomized Controlled Trial
Source: JMIR Res Protoc. 2021 Dec 3;10(12):e27462. doi: 10.2196/27462 (PMC8686407; doi:10.2196/27462)
Supplement: Multimedia Appendix 1 [file resprot_v10i12e27462_app1.docx]

Multimedia Appendix 1. Standard Protocol Items: Recommendations for International Trials (SPIRIT) figure

|  |  | **STUDY PERIOD** | | |
| --- | --- | --- | --- | --- |
|  | **Enrolment** | **Allocation** | **Post-allocation** | |
| **TIMEPOINT** | ***-t_1_*** | **0** | ***t_1_ Post measurement*** | ***t_2_ 4 week Follow-up*** |
| **ENROLMENT:** |  |  |  |  |
| **Eligibility screen** | X |  |  |  |
| **Informed consent** | X |  |  |  |
| **Randomization** |  | X |  |  |
| **INTERVENTIONS:** |  |  |  |  |
| ***EMIcompass + TAU*** |  |  |  |  |
| ***TAU*** |  |  |  |  |
| **ASSESSMENTS:** |  |  |  |  |
| **SCREENING** |  |  |  |  |
| ***SCID-5*** | X |  | X | X |
| ***CAARMS past 4 weeks*** | X |  |  |  |
| ***BPRS*** | X |  |  |  |
| ***SOFAS*** | X |  |  |  |
| ***HAM-D*** | X |  |  |  |
| ***HAM-A*** | X |  |  |  |
| ***YMRS*** | X |  |  |  |
| ***Fam. Risk*** | X |  |  |  |
| ***K10*** | X |  |  |  |
| ***PQ-SV*** | X |  |  |  |
| ***SPQ*** | X |  |  |  |
| ***Stress reactivity*** | X |  | X | X |
| ***PSRS*** | X |  | X | X |
| ***PANAS*** | X |  | X | X |
| **PRIMARY CANDIDATE MECHANISM** |  |  |  |  |
| ***EMA: stress reactivity*** |  | X | X | X |
| **SECONDARY CANDIDATE MECHANISM** |  |  |  |  |
| ***EMA: threat anticipation*** |  | X | X | X |
| ***EMA: negative affective appraisals*** |  | X | X | X |
| ***EMA: resilience to stress*** |  | X | X | X |
| ***TAM*** |  | X | X | X |
| ***IPSM*** |  | X | X | X |
| ***CD-RISC*** |  | X | X | X |
| ***RS-13*** |  | X | X | X |
| ***ECG markers of stress sensitivity*** |  | X | X | X |
| ***PRIMARY OUTCOME*** |  |  |  |  |
| ***K10*** |  | X | X | X |
| ***SECONDARY OUTCOME*** |  |  |  |  |
| ***CAARMS since last assessment*** |  |  | X | X |
| ***BPRS*** |  |  | X | X |
| ***SOFAS*** |  |  | X | X |
| ***HAM-D*** |  |  | X | X |
| ***HAM-A*** |  |  | X | X |
| ***YMRS*** |  |  | X | X |
| ***PQ*** |  |  | X | X |
| ***BDI-II*** |  | X | X | X |
| ***BSI*** |  | X | X | X |
| ***WHOQOL-BREFf*** |  | X | X | X |
| ***FFMQ*** |  | X | X | X |
| ***SCS*** |  | X | X | X |
| ***CERQ*** |  | X | X | X |
| **ACCEPTABILITY MEASURES** |  |  |  |  |
| ***EMA debriefing*** |  | X | X | X |
| ***Debriefing intervention*** |  |  | X |  |
| ***WAI-T*** |  |  | X |  |
| ***WAI-P*** |  |  | X |  |
| **OTHER** |  |  |  |  |
| ***CSRI*** | X |  | X | X |
| ***WPAI*** | X |  | X | X |
| ***Medication use*** | X |  |  |  |
| ***CTQ*** |  | X |  |  |

*Notes:* Structured Clinical Interview for DSM-5 (SCID-5; 79); Social and Occupational Functioning Assessment Scale (SOFAS;84); Comprehensive Assessment of At Risk Mental State (CAARMS; 80); Brief Psychiatric Rating Scale (BPRS;104) ; Brief Symptom Inventory (BSI-18; 106); Beck Depression Inventory-II (BDI-II; 107); Hamilton Depression Rating Scale (HAM-D; 85); Hamilton Anxiety Rating Scale (HAM-A; 86); Young Mania Rating Scale (YMRS; 105); Kessler Distress Scale (K-10; 81); Positive Affect Negative Affect Scale (PANAS; ^77^); Stress reactivity assessed using two items; Threat Anticipation Measure (TAM; 62); Interpersonal Sensitivity Measure (IPSM; 98); Connor-Davidson Resilience Scale (CD-RISC; 99); Resilience scale (RS-13; 100); Self-compassion Scale (SCS; 101); Five Facet Mindfulness Questionnaire (FFMQ; 102); Working Alliance Inventory – Short form (WAI-SF; 111, 112); Childhood Trauma Questionnaire (CTQ; 110); Prodromal Questionnaire – Short version (PQ-SV; 108) ; Schizotypal Personality Questionnaire (SPQ; 124); Cognitive Emotion Regulation Questionnaire (CERQ; 103); Client Service Receipt Inventory (CSSRI; 87); Work Productivity and Activity Impairment questionnaire (WPAI; 125); WHO-Quality of Life (WHOQOL-BREF; 109); Ecological Momentary Assessment (EMA); Electrocardiography (ECG).

**References:**

124 Raine, A & Benishay, D J. The SPQ-B: A brief screening instrument for schizotypal personality disorder. Journal of Personality Disorders 1995; 9: 346-355 [doi: [10.1521/pedi.1995.9.4.346](https://doi.org/10.1521/pedi.1995.9.4.346)]

125 Reilly, M C, Zbrozek, A S & Dukes, E M. The validity and reproducibility of a work productivity and activity impairment instrument. PharmacoEconomics 1993; 4: 353-365 [doi:10.2165/00019053-199304050-00006]
